# Molecular matched targeted therapies for primary brain tumors—a single center retrospective analysis

**DOI:** 10.1007/s11060-022-04049-w

**Published:** 2022-07-21

**Authors:** Anna-Luisa Luger, Sven König, Patrick Felix Samp, Hans Urban, Iris Divé, Michael C. Burger, Martin Voss, Kea Franz, Emmanouil Fokas, Katharina Filipski, Melanie-Christin Demes, Albrecht Stenzinger, Felix Sahm, David E. Reuss, Patrick N. Harter, Sebastian Wagner, Elke Hattingen, Jennifer Wichert, Constantin Lapa, Stefan Fröhling, Joachim P. Steinbach, Michael W. Ronellenfitsch

**Affiliations:** 1 Dr. Senckenberg Institute of Neurooncology, University Hospital Frankfurt, Goethe University, Frankfurt am Main, Germany; 2 German Cancer Consortium (DKTK), Partner Site Frankfurt/Mainz, Frankfurt am Main, Germany; 3 Frankfurt Cancer Institute (FCI), University Hospital Frankfurt, Goethe University, Frankfurt am Main, Germany; 4 University Cancer Center Frankfurt (UCT), University Hospital Frankfurt, Goethe University, Frankfurt am Main, Germany; 5 Department of Neuroradiology, University Hospital Frankfurt, Goethe University, Frankfurt am Main, Germany; 6 Department of Neurosurgery, University Hospital Frankfurt, Goethe University, Frankfurt am Main, Germany; 7 Department of Radiotherapy and Oncology, University Hospital Frankfurt, Goethe University, Frankfurt am Main, Germany; 8 Neurological Institute (Edinger Institute), University Hospital Frankfurt, Goethe University, Frankfurt am Main, Germany; 9 German Cancer Research Center (DKFZ), Heidelberg, Germany; 10 Dr. Senckenberg Institute of Pathology, University Hospital Frankfurt, Frankfurt am Main, Germany; 11 Institute of Pathology, University Hospital Heidelberg, Heidelberg, Germany; 12 Centers for Personalized Medicine (ZPM), Heidelberg Site, Heidelberg, Germany; 13 Department of Neuropathology, University Hospital Heidelberg, Heidelberg, Germany; 14 Clinical Cooperation Unit Neuropathology, Heidelberg, Germany; 15 German Cancer Consortium (DKTK), Heidelberg, Germany; 16 Department of Medicine, Hematology/Oncology, University Hospital Frankfurt, Goethe University, Frankfurt am Main, Germany; 17 Department of Nuclear Medicine, University Hospital Frankfurt, Goethe University, Frankfurt am Main, Germany; 18 Faculty of Medicine, Nuclear Medicine, University of Augsburg, Augsburg, Germany; 19 Department of Nuclear Medicine, University Hospital Würzburg, Würzburg, Germany; 20 Division of Translational Medical Oncology, National Center for Tumor Diseases (NCT) and German Cancer Research Center (DKFZ), Heidelberg, Germany

**Keywords:** Brain tumor, Glioma, Molecular matched therapy, Targeted therapy, Molecular profiling

## Abstract

**Purpose:**

Molecular diagnostics including next generation gene sequencing are increasingly used to determine options for individualized therapies in brain tumor patients. We aimed to evaluate the decision-making process of molecular targeted therapies and analyze data on tolerability as well as signals for efficacy.

**Methods:**

Via retrospective analysis, we identified primary brain tumor patients who were treated off-label with a targeted therapy at the University Hospital Frankfurt, Goethe University. We analyzed which types of molecular alterations were utilized to guide molecular off-label therapies and the diagnostic procedures for their assessment during the period from 2008 to 2021. Data on tolerability and outcomes were collected.

**Results:**

413 off-label therapies were identified with an increasing annual number for the interval after 2016. 37 interventions (9%) were targeted therapies based on molecular markers. Glioma and meningioma were the most frequent entities treated with molecular matched targeted therapies. Rare entities comprised e.g. medulloblastoma and papillary craniopharyngeoma. Molecular targeted approaches included checkpoint inhibitors, inhibitors of mTOR, FGFR, ALK, MET, ROS1, PIK3CA, CDK4/6, BRAF/MEK and PARP. Responses in the first follow-up MRI were partial response (13.5%), stable disease (29.7%) and progressive disease (46.0%). There were no new safety signals. Adverse events with fatal outcome (CTCAE grade 5) were not observed. Only, two patients discontinued treatment due to side effects. Median progression-free and overall survival were 9.1/18 months in patients with at least stable disease, and 1.8/3.6 months in those with progressive disease at the first follow-up MRI.

**Conclusion:**

A broad range of actionable alterations was targeted with available molecular therapeutics.

However, efficacy was largely observed in entities with paradigmatic oncogenic drivers, in particular with *BRAF* mutations. Further research on biomarker-informed molecular matched therapies is urgently necessary.

**Supplementary Information:**

The online version contains supplementary material available at 10.1007/s11060-022-04049-w.

## Introduction

Primary CNS tumors comprise a heterogeneous group of benign and malignant tumors. Glioblastoma (GB) is the most common entity in the group of primary malignant brain tumors and characterized by pronounced therapy resistance and poor prognosis. While a standard first line treatment has been defined for various primary brain tumors, general standards for treatment at recurrence are mostly lacking [1–4]. In recent years, various studies have attempted to evaluate new therapeutic strategies including anti-angiogenic approaches, immunotherapies, and molecular targeted strategies in the first line and recurrent disease setting, but delivered overall disappointing results [5–14].

Due to a lack of therapeutic options, especially for patients with relapsing tumors in good clinical condition, individual off-label therapies can be considered and reimbursement can be granted by health insurances. Decision-making on the drug of choice usually involves discussion in neurooncological and/or molecular tumor boards and is guided by (preliminary) results of clinical trials or specific molecular markers. An example of trial-based guidance in GB therapy is regorafenib in recurrent disease according to a positive phase II trial despite the lack of confirmation of efficacy in a phase III trial [15]. A prime example for an individualized concept in neurooncology is the NCT neuro master match (N2M2; NOA20) study in which the molecular signature of GBs determines the treatment arm [16]. In line with the trend towards individualized therapy in clinical trials, molecular marker guided decision making is frequently used for off-label therapies and has to some degree replaced the “one size fits all” approach [17].

For many brain tumor entities, frequent activation of specific signaling pathways has been demonstrated [18]. Because such activation may be due to a spectrum of different genetic alterations more comprehensive genetic analyses can be helpful and may also help diagnostically in unclear cases. One trial applying multidimensional characterization of tumors using whole-genome/exome and RNA sequencing to reveal targeted therapeutic strategies in younger patients is the molecularly aided stratification for tumor eradication research (MASTER) program of the National center for tumor diseases (NCT) and the German Cancer Consortium (DKTK) [19, 20].

For several tumor entities like malignant gliomas, certain molecular analyses are already part of the standard pathology workup due to their role in diagnostics (e.g. *1p19q* codeletion to confirm oligodendroglioma or EGFR amplification as a novel criterion for GB) or their impact on prognosis and response to treatment (e.g. *MGMT* promoter methylation status to determine temozolomide efficacy). For specific rare brain cancer entities, molecular matched therapies targeting key driver mutations have already produced encouraging results. Examples are *BRAF* mutations in pleomorphic xanthoastrocytoma (PXA) and papillary craniopharnygeoma (PCP) as well as *TSC* mutations in subependymal giant cell astrocytoma (SEGAs) that can be targeted by BRAF or mTOR inhibitors (e.g. vemurafenib or everolimus) [21–25].

To evaluate our decision-making process and potential efficacies of off-label molecular matched targeted therapies, we performed a retrospective analysis to determine which agents were used based on which grounds to treat primary brain tumors during the period from 2008 to 2021. Additionally, we collected data on tolerability and signals for efficacy.

## Material and methods

### Study population and statistical analysis

**Fig. 1:**
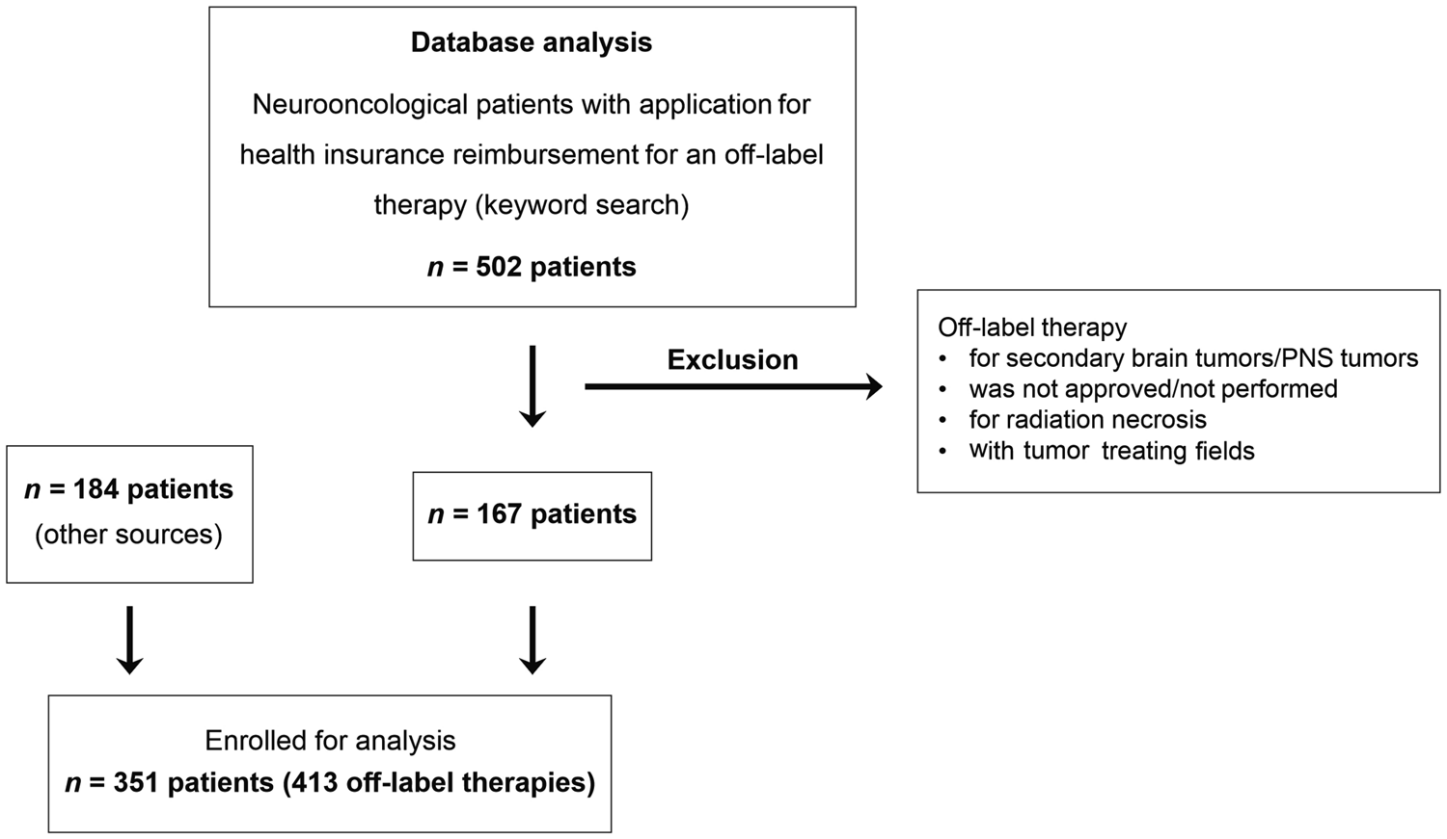
Patient selection of the current study. The clinical database of our university healthcare center and the institutional server were scanned for neurooncological patients for who reimbursement requests for off-label therapies had been drafted between January 2008 and April 2021

A retrospective case analysis was performed to identify off-label medications employed in the treatment of brain tumors in adults from 2008 to 2021 (Fig. 1). Ethics approval for this analysis was granted by our institutional review board (ethics committee at the University Hospital Frankfurt; reference number SNO-3-2021).

The clinical database of our university healthcare center was scanned for neurooncological patients for whom an application for health insurance reimbursement for an off-label therapy had been submitted between January 2008 and April 2021. Prior to implementation of our digital hospital patient management system, patient records for off-label interventions were stored as print out documents or single files on the hospital server. These data were analyzed manually (“other sources”). Applications from later years were saved in our digital system and were scanned electronically and then manually reanalyzed. Exclusion criteria were secondary brain tumors (brain metastases or meningeosis neoplastica), tumors of the peripheral nervous system, tumor treating fields as off-label therapies, and patients who received an individual off-label therapy whose purpose was not anti-tumor therapy, e.g. bevacizumab to treat radiation necrosis. Identified off-label therapies were divided into the subgroups: treatment in analogy to (ongoing) clinical trials or off-label molecular matched targeted therapy. Off-label therapies in analogy to clinicial trials were not further analyzed in this manuscript. Subsequently, data on the specifics as well as number of individual off-label therapies per case were collected and analyzed from date of initial diagnosis until death, the last contact or the end of the follow-up period in December 2021.

### Magnetic resonance imaging

For MRI follow-up, all patients had at least T1-weighted (T1-w) sequences before and after intravenous administration of Gadolinium-containing contrast agent and T2-w sequences on a 1.5 or 3 Tesla MRI scanner in a radiological practice or the Department of Neuroradiology, University Hospital Frankfurt. The first MRI was performed in median after 6 weeks after the initiation of therapy and was assessed according to RANO criteria by an experienced neuroradiologist (P.S.) [26]. Additionally, the date of tumor progression under treatment was determined.

### Immunohistochemistry

Immunohistochemistry (IHC) for all targets was performed using standard protocols on the automated immunohistochemistry staining system Discovery XT (Roche/Ventana, Tucson, Arizona, USA) and the LEICA BOND-III automated stainer (Leica, Wetzlar, Germany) respectively. The following antibodies were used: PD-L1 (Cell Signaling, Boston, U.S.A.), p-S6K1 (Cell Signaling, Boston, U.S.A.), p-4EBP1 (Cell Signaling, Boston, U.S.A.), p-RPS6 (Ser 235/236 and Ser240/244, Cell Signaling, Boston, U.S.A.), p-PRAS40 (Cell Signaling, Boston, U.S.A.), p-NDRG1 (Cell Signaling, Boston, U.S.A.), p-mTOR (S2448, Cell Signaling, Boston, U.S.A.), IDH1_R132H (Dianova, Eching, Germany) and BRAF V600E (DCS, Hamburg, Germany) [27].

### Detection of ﻿*BRAF V600* mutations by real-time PCR

In patients 13 and 15 mutations in the *BRAF* gene targeting the amino acid valine at position 600 of the protein were detected with the AmoyDx®BRAF V600 Mutations Detection Kit (Amoy Diagnostics, Co., Ltd., Xiamen, China). The analysis was performed with DNA extracted from formalin-fixed paraffin-embedded tumor tissue according to the protocol provided by the manufacturer. The real-time PCR assay uses the amplification refractory mutation system (ARMS) technology and covers the following ﻿*BRAF V600* mutations (base exchanges; Cosmic ID): V600E (1799T > A; 476), V600K (1798_1799GT > AA; 473), V600E2 (1799_1800TG > AA; 475), V600R (1798_1799GT > AG; 474), V600D (1799_1800TG > AC; /) and V600D2 (1799_1800TG > AT; 477).

### Detection of *BRAF V600* mutations by single gene sequencing with pyrosequencing

In, patients 12 and 26, single gene sequencing with pyrosequencing (PyroMark Q24, QIAGEN) for detection of *BRAF V600E* mutations was performed using the Therascreen BRAF Kit from QIAGEN at the Department of Pathology, University Hospital Frankfurt.

### Human methylation EPIC array

Tumor DNA was isolated from representative FFPE tissue. DNA was further processed and hybridized to the Human Methylation EPIC array beadchips (Illumina, California, USA) following protocols provided by the manufacturer. EPIC array beadchips were scanned by an iScan (Illumina, California, USA) and raw intensity data (idats) was obtained for upload to the website molecularpathology.org provided by the University of Heidelberg, Germany. Calibrated scores for DNA methylation classes and subclasses, copy number variation profiles and MGMT promoter methylation status were recorded (MolecularNeuroPathology.org 2018–Version 3.1.5).

### NGS panel sequencing

For patients 3, 9, 18, 19 and 28 NGS panel sequencing was performed on a NextSeq 500 instrument (Illumina) as previously described at the Department of Neuropathology, University Hospital Heidelberg [28]. In brief, a capture-based custom brain tumor panel (Agilent Technologies, Santa Clara, CA, USA) was used covering the entire coding and selected intronic and promoter regions of genes of particular relevance in CNS tumors (130 genes in NPHD 2015 and 171 genes in NPHD 2019).

For patients 11 and 14 DNA and RNA based NGS panel sequencing was performed on a GeneReader Platform (QIAGEN) by using the nNGM V1.0 Panel and the QIAact RNA Fusion UMI Panel Kit (Qiagen) at the Department of Pathology, University Hospital Frankfurt. By focusing on clinically meaningful mutations the QIAGEN clinical insight analyze (QCIA) und QIAGEN clinical insight (QCI) Interpret were applied (reference genome hg19).

For patients 16, 17 and 24 hybrid capture-based panel-sequencing was performed as previously described at the Institute of Pathology, University Hospital Heidelberg [29, 30]. Briefly, after library preparation for the capture-based TruSight Oncology 500 panel (Illumina), DNA integrity assessment fragmentation, enriched libraries were amplified and sequenced on a NextSeq 500 instrument (Illumina). All assays were performed according to the manufacturers’ protocols. Processing of raw sequencing data and variant calling was carried out using the TruSight Oncology 500 Local App (version 1.3.0.39). Called variants were verified by visual inspection in the Integrative Genomics Viewer [31]. Only variants with an allele frequency above 2% and a minimum coverage of greater than × 100 were considered [30].

### NCT master and exome sequencing

Patients 25 and 29 were studied by whole-exome and RNA sequencing within the MASTER program, a prospective observational study by NCT and DKTK that enrolls younger adults with advanced cancers across entities and adult patients with advanced rare malignancies across age groups [19, 20]. Patient 27 was studied by whole-exome sequencing.

### DOTATOC-PET

For patients 20–22: The somatostatin analogue DOTATOC (DOTA-D-Phe1-Tyr3- octreotide) was labeled with 68 Ga eluted from an in-house Ge68/Ga68 generator as described [32–34]. 68 Ga DOTATOC-PET/CT was performed on a hybrid PET/CT scanner (Biograph 6, Siemens medical Solutions Inc., Hoffman Estates, Illinois, USA) according to standard protocols [35].

### Statistical analysis

Progression-free (PFS) and overall survival (OS), defined as the time from initiation of the individual off-label treatment until progression or death from any cause, was determined using Kaplan–Meier analysis for the first off-label therapy of each patient and additionally for each off-label therapy and (Suppl. Figure 1). Statistical significance between the two subgroups of patients with at least stable disease (SD) (including pseudoprogression, and responses) and progressive disease (PD) in the first MRI after start of treatment was calculated by univariate analysis using the log-rank (Mantel-Cox) test. A p-value of < 0.05 was considered statistically significant (Graph Pad Prism 5.0, GraphPad Software, Inc., San Diego, CA, USA).

## Results

### Identification of a brain tumor cohort treated with off-label therapies

**Fig. 2:**
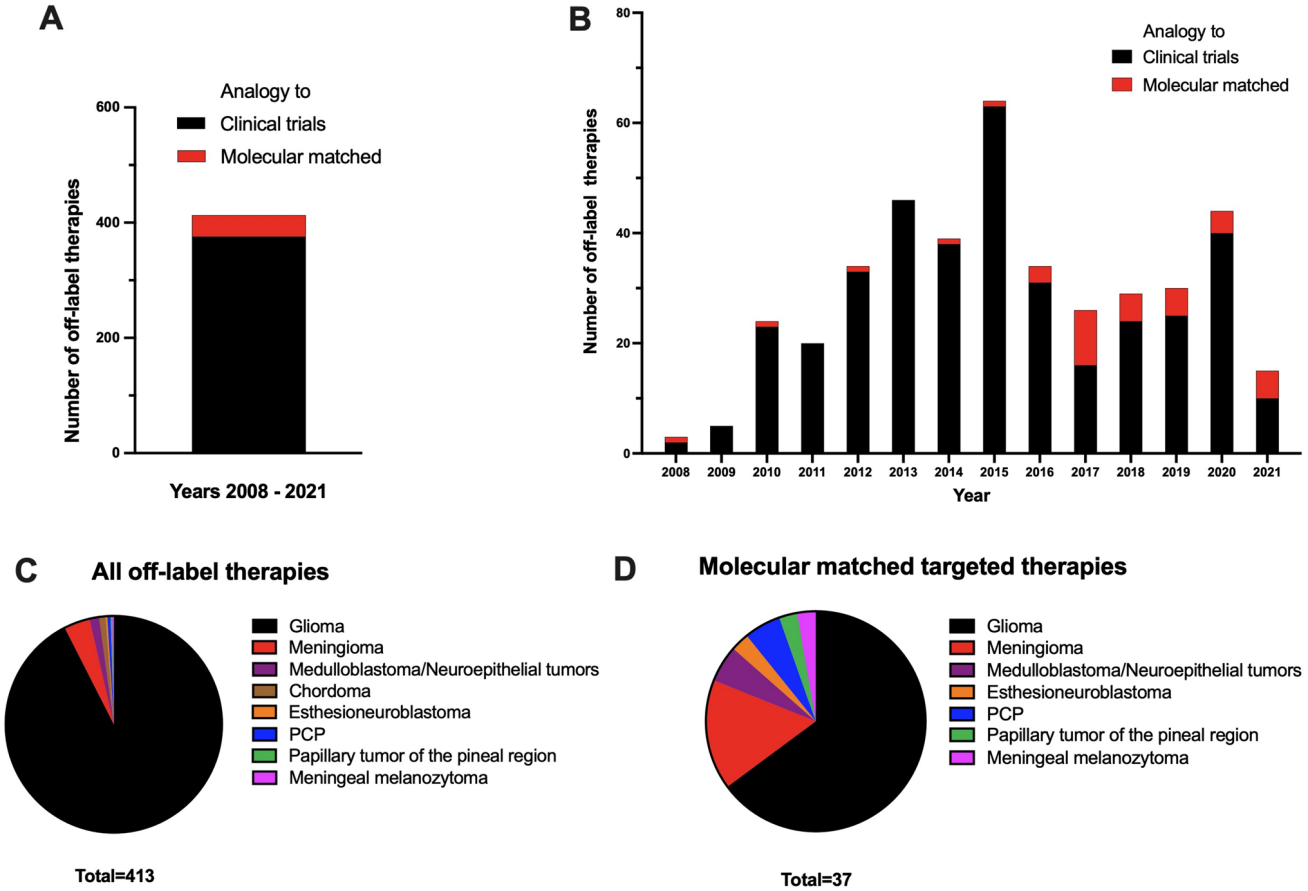
Characteristics of the patient cohort. **A** 413 off-label therapies (351 patients) were identified from January 2008 to April 2021. 376 (91%) of these procedures were carried out in analogy to clinical trials. 37 (9%) therapies were performed as molecular matched targeted therapies. **B** shows the number of off-label therapies per year. The two subgroups "Clinical Trials" and "Molecular Matched" are shown in contrasting colors. C/D: the pie charts show the entities of the entire cohort (**C**) and the cohort of patients with a molecular matched targeted therapy (**D**). PCP: papillary craniopharnygeoma

A total of 413 off-label therapeutic interventions were identified in 351 patients, 376 (91%) therapies were performed in analogy to preliminary positive data from clinical trials, and 37 (9%) therapies were performed as molecular matched targeted therapies (Fig. 2A). In a year-by-year analysis, molecular matched therapies were administered with an increasing proportion from 2016 compared to previous years. (Fig. 2B). While the proportion of gliomas in the entire cohort was 92%, the proportion of gliomas in the group with molecular matched therapies was lower with 65% (Fig. 2C–D).

### Characteristics of patients receiving a molecular matched therapy

Altogether 37 molecular matched therapies were applied in 29 patients. 23/29 patients received one, 4/29 received two and 2/29 patients received three different molecular matched targeted therapies (Table 1). 28% of the patients (8/29) were female. Patients were a median of 47 years old when off-label therapy was initiated (range 19–81). In median, patients had received three prior treatments before the molecular matched therapy was started (range 1–10). In 6/37 off-label-therapies conventional treatments (mainly radio- and/or chemotherapy) were administered in parallel.

**Table 1:** Patient characteristics of patients receiving a molecular matched targeted therapy. Characteristics of patients receiving a molecular matched targeted therapy are shown. Patient 1 received radiochemotherapy with temozolomide plus lomustine in addition to nivolumab. Patient 4 received chemotherapy with temozolomide plus lomustine. References for literature corroborating the therapy decisions are attached. Combined treatment approaches (e.g. resection followed by radio- plus chemotherapy) were counted as one therapy unit

Pat	Age	Sex	Histology	Total number of prior therapies	Targeted therapy	Combination with other therapies	Molecular marker	Method	Time between marker detection and start of therapy	Number of therapies between marker detection and start of therapy
1	46, 47	m	GB, IDH-wt (CNS WHO grade 4)	1, 4	Nivolumab [9], Nivolumab/Bevacizumab [36]	RCHT, –	PD-L1 (PD-L1 score: 8)	IHC	1, 13	0, 3
2	62	m	GB, IDH-wt (CNS WHO grade 4)	3	Nivolumab [9]	–	PD-L1 (PD-L1 score: 4)	IHC	7	2
3	59	f	GB, IDH-wt (CNS WHO grade 4)	3	Nivolumab [9]	–	Hypermutator phenotype with *MLH-1* mutation (TMB 30/MB, AF (MLH-1): 87%, TCC: ≥ 70%)	Panel sequencing	10	2
4	19	m	Diffuse hemispheric glioma, H3 G34-mutant (CNS WHO grade 4)	0	Nivolumab [9]	CHT	PD-L1 (PD-L1 score: 6–9)	IHC	3	0
5	57	m	Malignant glioma, NOS	4	Nivolumab [9]	–	PD-L1 (PD-L1 score: 8–12)	IHC	23	3
6	44	m	GB, IDH-wt (CNS WHO °4)	1	Nivolumab [9]	RT/TTF	PD-L1 (PD-L1 score: 12)	IHC	1	0
7	59	m	GB, IDH-wt (CNS WHO °4)	3	Temsirolimus [14, 16]	–	p-4EBP1, p-S6RP, p-PRAS40, p-NDRG1, P-mTOR	IHC	13	3
8	46	m	GB, IDH-wt (CNS WHO °4)	6	Everolimus/Bevacizumab [37, 38]	–	P-S6K1	IHC	6	1
9	31	m	GB, IDH-wt (CNS WHO °4)	3	Palbociclib [16]	–	Homozyg. deletion of *CDKN2A/B *(TCC: ≥ 70%)	Panel sequencing	12	3
10	48	m	GB, IDH-wt (CNS WHO grade 4)	3	Palbociclib [16]	RT	Amplification of *CDK4* and homozygous deletion of *CDKN2A*	850 k array	11	3
11	56, 57	m	Malignant Glioma, BRAF-altered PXA suspected	3, 4	Dabrafenib [39, 40], Dabrafenib/Trametinib [41]	–, Chloroquine	*BRAF V600E* mutation (AF: 12%)	IHC/Panel sequencing	7, 16	1, 3
12	27, 27	m	Malignant Glioma, BRAF-altered PXA suspected (suspicious for leptomeningeal disease)	3, 4	Dabrafenib [39, 40], Dabrafenib/Trametinib [41]	–, Chloroquine	*BRAF V600E *mutation (AF: 42%)	IHC/Pryosequencing	6, 10	2, 3
13	25, 30, 31	m	PXA (CNS WHO grade 3) with leptomeningeal disease	2, 3, 4	Dabrafenib [39, 40], Dabrafenib/Trametinib [41], Binimetinib/Encorafenib [42]	–, –, –	*BRAF V600E* mutation	IHC/RT PCR	7, 58, 71	2, 3, 4
14	42	m	PXA (CNS WHO grade 3) (suspicious for leptomeningeal disease)	4	Dabrafenib/Trametinib [43, 44]	–	*BRAF non-V600E* mutation (AF: 14%, TCC: 60%)	Panel sequencing	n.a	n.a
15	50	f	Malignant glioma, BRAF-altered, NOS	5	Dabrafenib/Trametinib [41]	–	*BRAF V600E *mutation	IHC/RT PCR	47	2
16	49	f	Oligodendroglioma, IDH-mutant and 1p719q codeleted (CNS WHO grade 3)	8	Alpelisib/ketogenic diet [45]	–	Activating *PIK3CA* mutation (AF: 32.8%, TCC: 90%)	Panel sequencing	13	2
17	25	f	Diffuse midline glioma H3 K27-altered (CNS WHO grade 4)	1	Pemigatinib [46, 47]	–	*FGFR1* mutation (AF: 77.1%, TCC: 100%)	Panel sequencing	4	1
18	46	m	Diffuse midline glioma H3 K27-altered (CNS WHO grade 4)	2	Pemigatinib [46, 47]	–	*FGFR1* mutation (AF: approx. 25%, TCC: approx. 60–70%)	Panel sequencing	19	2
19	24	f	Supratentorial ependymoma (CNS WHO grade 3)	3	Trametinib [48]	–	*BRAF K601E *mutation (AF: 45%, TCC: ≥ 70%)	Panel sequencing	6	0
20	57	m	Meningioma (CNS WHO grade 2)	5	Sandostatin [49]	–	Somatostatin rezeptor	DOTATOC-PET	0	0
21	39, 40, 40	m	Meningioma (CNS WHO grade 3)	8, 9, 10	PRRT [50], Everolimus [51–53], Everolimus/Bevacizumab [54]	–, –, –	Somatostatin rezeptor p-4EBP1, p-S6-RP, pPRAS40	DOTATOC-PET IHC	1, 20, 23	0, 5, 6
22	81	m	Meningioma (CNS WHO grade 3)	5	Sandostatin [49]	–	Somatostatin rezeptor	DOTATOC-PET	2	0
23	72	f	Meningioma (CNS WHO grade 3)	6	Everolimus [53]	–	P-S6RP	IHC	5	1
24	78	m	Medulloblastoma (CNS WHO grade 4)	5	Olaparib [55]	–	*KMT2C* mutation (AF: 23.8%, TCC: 60%)	Panel sequencing	56	5
25	35	m	Papillary tumor of the pineal region (CNS WHO grade 2)	3	Everolimus [56]	–	Allelic loss of *PTEN* and *FGFR1*amplification (TCN: 3.7); hyperactivation of the mTOR pathway	Whole-exome/genome and RNA sequencing (NCT Master), IHC	18	1
26	61, 61	m	PCP (CNS WHO grade 1)	2, 3	Dabrafenib/Trametinib [57], Vemurafeninb [58]	–, –	*BRAF V600E *Mutation (AF: 19%)	IHC/Pryosequencing	1, 4	1, 2
27	62	f	Esthesioneuroblastoma	8	Crizotinib [59]	–	Focal amplification of chromosome 15, *NTRK3* amplification (TCN: 3.8)	Exome sequencing	38	1
28	54	f	HGNET-MN1-altered	8	Everolimus [23, 60]	–	*TSC2* mutation (AF: 55%)	Panel sequencing	4	0
29	55	m	Meningeal melanocytoma	4	Trametinib [61]	–	*GNAQ* mutation (AF: 51%)	Whole-exome/genome and RNA sequencing (NCT Master)	72	0

AF: Allele frequency, GB: glioblastoma, HGNET-MN1-altered: High-grade neuroepithelial tumor with MN1 alteration, NOS: Not otherwise specified, PCP: papillary craniopharnygeoma, PRRT: peptide receptor radionuclide therapy, PXA: Pleomorphic xanthoastrocytoma, R(CH)T: radio(chemo)therapy, TCC: Tumor cell content, TTF: tumor treating fields, TCN: Total copy number, TMB: Tumor mutation burden

### Methods of molecular marker detection

Most molecular markers as basis for a targeted therapy were identified by immunohistochemistry, namely detection of PD-L1 expression, mTOR signaling activation via staining for phosphorylated target proteins and detection of BRAF V600E alterations via mutation specific antibodies (Table 1). Comprehensive molecular profiling (in most cases gene panel sequencing, in one case whole-exome sequencing and in two cases whole-exome as well as RNA sequencing within the NCT MASTER program) was also frequently applied for molecular diagnostics (Table 1). The expression of the somatostatin receptor was non-invasively analyzed by DOTATOC-PET (Table 1). Using Human Methylation EPIC array, amplification of *CDK4* and a homozygous deletion of *CDKN2A* were identified (Table 1).

### Signals for efficacy of molecular matched therapies

**Fig. 3:**
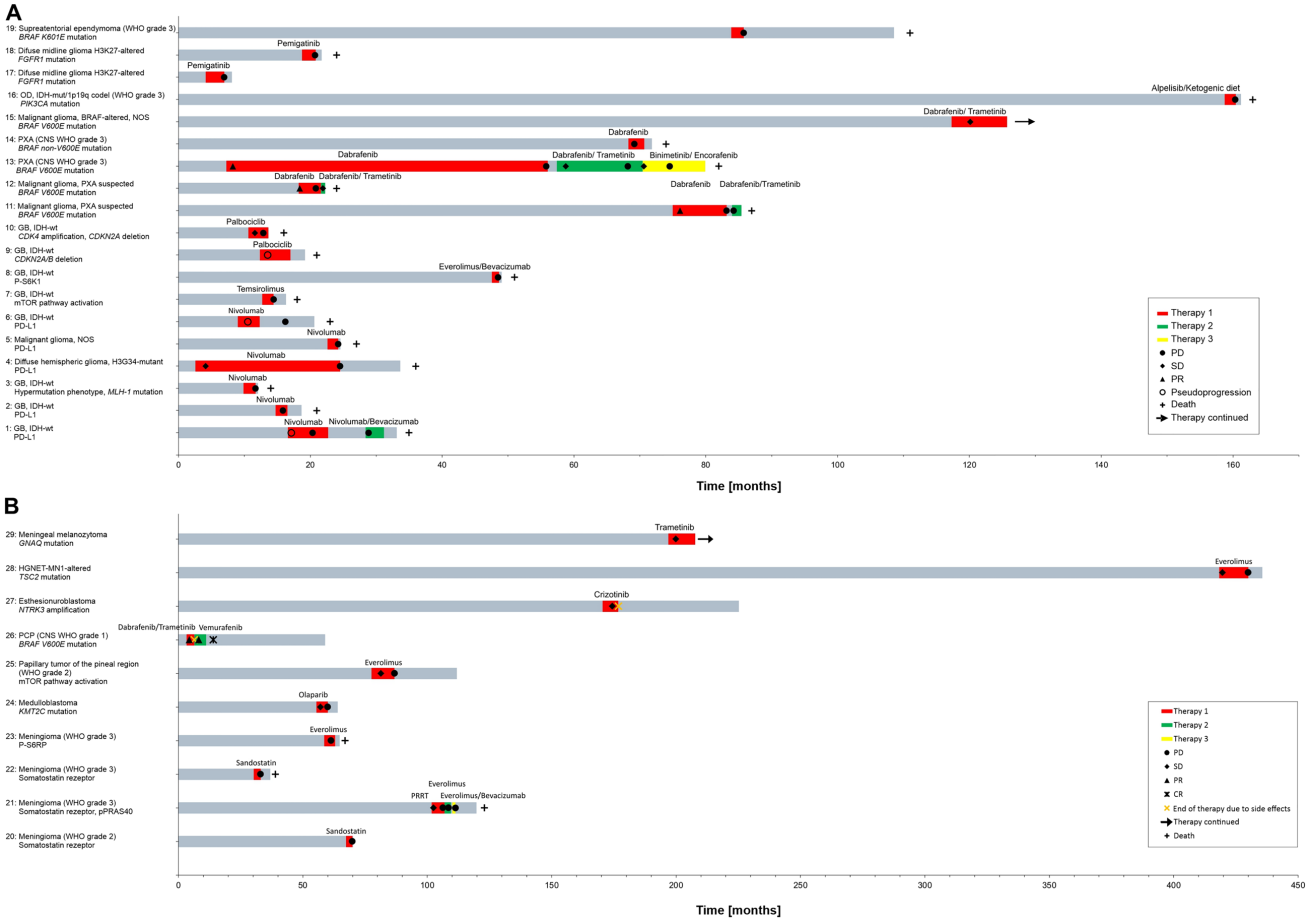
Course of treatment of brain tumor patients under molecular matched targeted therapies. **A**, **B** swimmer plot depicting treatment duration of molecular matched therapies as well as responses and disease progression for glioma patients (**A**; n = 19) and other entities (**B**; n = 10). Abbreviations: OD: Oligodendroglioma; NOS: Not otherwise specified; PXA: Pleomorphic xanthoastrocytoma; GB: Glioblastoma; PCP: papillary craniopharnygeoma; HGNET-MN1- altered: High-grade neuroepithelial tumor with MN1 alteration; PRRT: peptide receptor radionuclide therapy

**Fig. 4:**
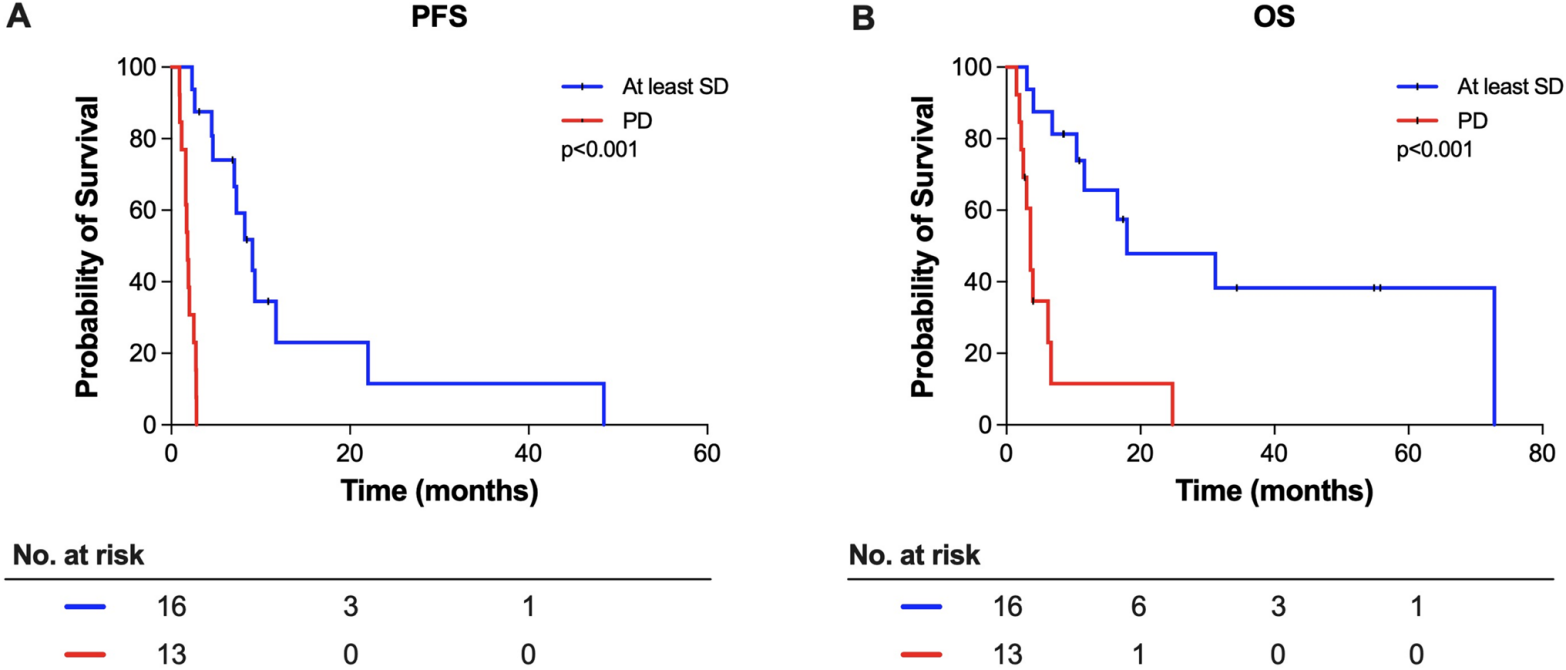
Survival of brain tumor patients under molecular matched targeted therapies. **A**/**B** Progression free survival (PFS) and overall survival (OS) of patients with at least stable disease (SD) and of patients with progressive disease (PD) treated with a molecular matched therapy. Only the the first molecular matched therapy of each patient is calculated. Tick marks indicate censored patients

Treatment duration of gliomas (Fig. 3A) and other entities (Fig. 3B) treated with a molecular matched therapy ranged from one to 49 months and complete response to PD respectively. In the first MRI after start of treatment, 46.0% of the cases showed PD (17/37), 29.7% SD (11/37), 13.5% partial response (PR) (5/37), and 10.8% (4/37) of the MRIs were suspicious for pseudoprogression (3/37) or not assessable (1/37) (Fig. 3). Unfortunately, historical controls for rare tumor entities are lacking. However, we and others have already shown that MRI response correlates not only with PFS but also with OS [62, 63]. Therefore, two groups were generated: One group containing all patients with at least stable diseases at first MRI and another group with PD at first MRI. When only evaluating the first off-target therapy for each patient median PFS of patients with at least SD and of patients with PD in the first follow-up MRI were 9.1 and 1.8 months, respectively (p < 0.0001, 95% CI). Median overall survival (OS) of patients with at least SD and of patients with PD were 18 months and 3.6 months, respectively (p < 0.0001, 95% CI) (Fig. 4A, B). When evaluating all off-target therapies results were almost identical (median PFS of the SD-/PD-cohort: 9.1/1.7 months, p < 0.0001, 95% CI; median OS of the SD-/PD-cohort: 18/4 months, p < 0.0001, 95% CI) (Suppl. Figure 1 A, B). Notably, the cohort of off-label interventions with a PD at first MRI contained a higher proportion of WHO Grade 4 tumors. Furthermore, 6/29 patients received two or three off-label interventions.

### Immune-checkpoint inhibition (ICI) with nivolumab as an off-label therapy in brain tumors

Molecular matched therapies included immune-checkpoint inhibitors (ICIs) for malignant gliomas with high PD-L1 expression or a hypermutator phenotype (patients 1–6). Except for patient 4 with diffuse hemispheric glioma who achieved a SD in the first MRI after start of treatment, all other patients displayed PD. Patient 4 received by far the longest treatment with nivolumab over 22 months. However, this patient (18 years old at the beginning of nivolumab) had a methylated *MGMT* status and ICI therapy was added concomitantly to first line therapy with lomustine and temozolomide (Table 1).

### mTOR inhibition as an off-label therapy in brain tumors

Patient 7 and 8 with GBs were treated with either temsirolimus or everolimus, respectively, to inhibit mTOR signaling which based on IHC signals was activated. Both patients had had extensive prior therapies (Table 1). In both cases, the first MRI after initiation of therapy revealed tumor progression and therapy was subsequently discontinued (Fig. 3A).

Patients 21 and 23 with meningioma CNS WHO grade 3 were also treated with everolimus followed by everolimus plus bevacizumab (patient 21), or everolimus alone (patient 23) due to mTOR pathway activation in Phospho-IHC for three/one and four months, respectively until tumor progression (Table 1, Fig. 3B) [53].

A patient with a papillary tumor of the pineal region (CNS WHO grade 2; patient 25) and mTOR pathway activation in Phospho-IHC potentially due to allelic loss of *PTEN* and *FGFR1* amplification was treated with everolimus with resulting SD until tumor progression after nine months (Fig. 3B, Table 1).

Patient 28 with a high-grade neuroepithelial tumor with *MN1* alteration (HGNET-MN1-altered) and a *TSC2* mutation was treated with everolimus with SD over twelve months (Fig. 3B, Table 1).

### BRAF/MEK inhibition off-label therapy in brain tumors

Patients 11–15 had BRAF-altered malignant gliomas or PXAs and were treated with a BRAF inhibitor with or without a MEK inhibitor. Especially patient 13 (PXA CNS WHO grade 3) who received three different combinational approaches of BRAF/MEK inhibitors (1. dabrafenib, 2. dabrafenib/trametinib, 3. binimetinib/encorafenib) showed a very long response to therapy with initially SD under all three procedures and treatment durations of 49/13/9 months until therapy was discontinued due to PD (Fig. 3A). Patients 11, 12 and 15 also had SD but shorter times to treatment failure (Fig. 3A). Patient 15 was still on therapy after ten months in December 2021 (Fig. 3A). Notably, patients 11–13 have been previously published in a case series [64]. However, at that time, follow-up had only extended until eight months under dabrafenib for patient 11, three months under dabrafenib for patient 12 and 27 months under dabrafenib for patient 13 [64]. The second therapy with dabrafenib in combination with trametinib (all patients) as well as the third therapy with binimetinib/encorafenib (patient 13) had not been reported thus far.

Patient 19 with an ependymoma CNS WHO grade 3 and a *BRAF K601E* mutation in the panel sequencing analysis was treated with trametinib for two months until tumor progression (Fig. 3B, Table 1).

**Fig. 5:**
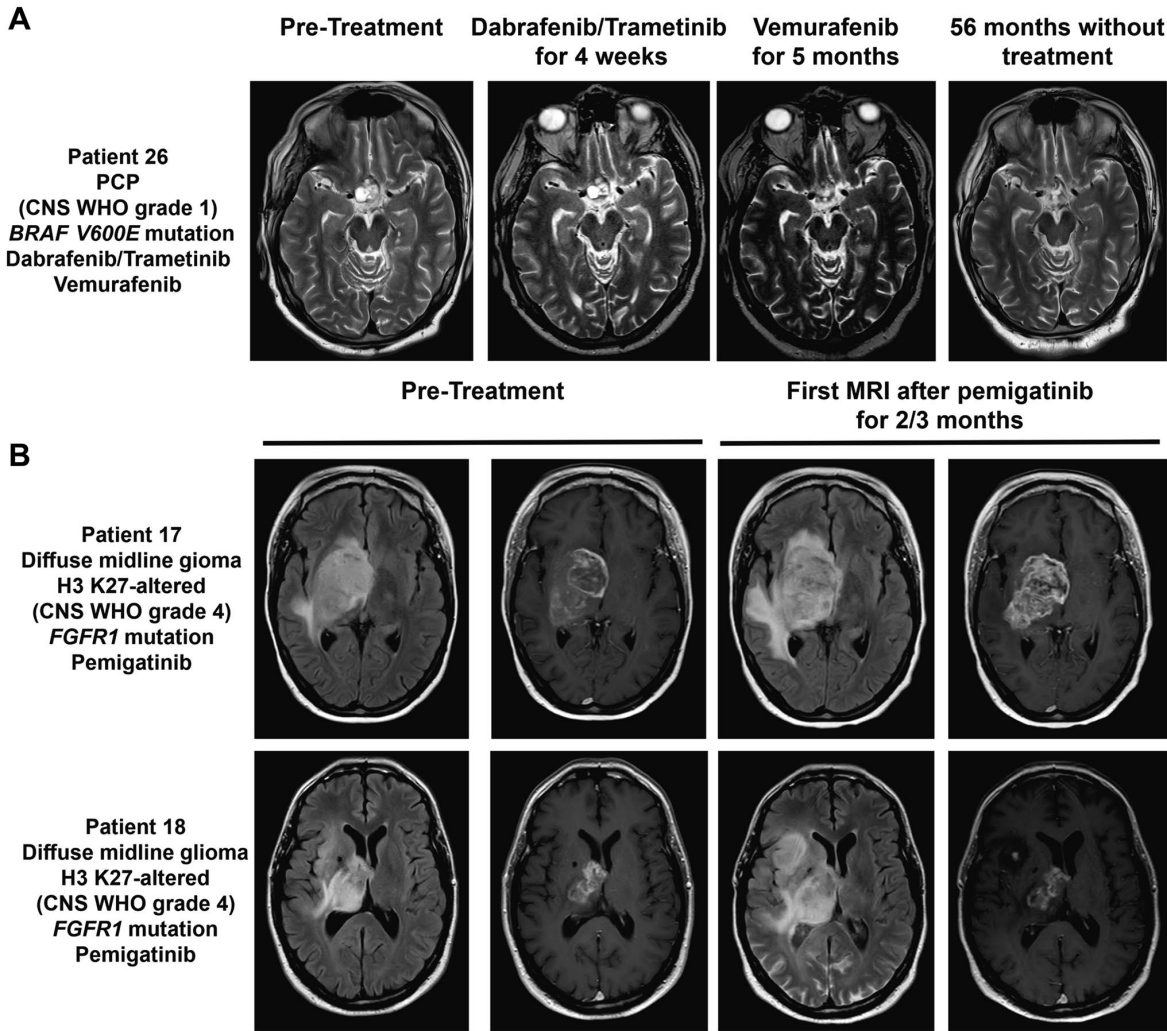
MRIs of selected cases. **A** axial T2-weightes magnetic resonance imaging sequences of patient 26 with a PCP with BRAF *V600E* mutation. First panel: after two tumor resections and one radiosurgery and before treatment with a BRAF inhibitor; second panel: after four weeks of treatment with dabrafenib/trametinib; third panel: after five months of treatment with vemurafenib (the patient was switched from dabrafenib/trametinib to vemurafenib due to side effects despite PR); fourth panel: after 56 months without therapy. **B** axial T2/fluid-attenuated inversion recovery-weighted magnetic resonance imaging sequences (left panel) and T1 post-contrast MRI (right panel) of two patients with recurrent H3K27-altered diffuse midline glioma and mutations in FGFR1 before and 2 months (patient 18)/ 3 months (patient 17) after start of treatment with pemigatinib

Patient 26 (PCP with *BRAF V600E* mutation) received a therapy with dabrafenib/trametinib, which was discontinued after three months despite a *partial response* on MRI because of sepsis-like episodes (Fig. 5A). The patient was switched to vemurafenib, which also caused serious side effects including sepsis and renal failure. After a brief pause in therapy, vemurafenib was continued at low dose. With a complete response on MRI, vemurafenib was discontinued after five months of therapy (Fig. 5B). After more than four years of follow-up without any tumor specific treatment, the patient remains recurrence-free.

A patient with meningeal melanocytoma and a *GNAQ* mutation (patient 29) was treated with the MEK inhibitor trametinib (level of evidence stage m1c) with SD in MRI after 3 months of treatment [61]. Therapy was still ongoing for twelve months (Fig. 3A, Table 1) at the end of the follow-up period.

### FGFR inhibition off-label therapy in diffuse midline gliomas H3K27-altered (CNS WHO grade 4)

Patient 17 and 18 with radiologically recurrent *H3K27*-altered diffuse midline glioma and *FGFR1* mutations were treated with the FGFR1/2/3 inhibitor pemigatinib. In patient 17, the identified mutation in *FGFR1* (*FGFR1:N456K*, likely pathogenic) had an allele frequency of 77.1% [65]. This *FGFR1* mutation has previously been reported in diffuse midline gliomas [66, 67]. In patient 18, the allele frequency of the identified mutation *FGFR1:N457D* (mutation rated as relevant) was approximately 25%. Both patients suffered from early tumor progression after three and two months, respectively (Fig. 3A, Fig. 5B).

### Sandostatin and peptide receptor radionuclide therapy as off-label therapy for somatostatin receptor expressing meningioma

Two patients with meningioma CNS WHO grade 2 and 3 (patient 20 and 22) were treated with sandostatin due to somatostatin receptor expression (as visualized by DOTATOC-PET) for two/three months until early tumor progression (Table 1, Fig. 3B).

Patient 21 with a meningioma CNS WHO grade 3 and somatostatin receptor expression was treated with peptide receptor radionuclide therapy with ^177^Lu-DOTATOC for five months until tumor progression [50]. Afterwards, everolimus and everolimus/bevacizumab were administered (Table 1, Fig. 3B).

### Other inhibitors of signal transduction as off-label therapy in brain tumors

Other off-label therapies included two GB patients with amplification of *CDK4* and/or a homozygous deletion of *CDKN2A* treated with the CDK4/6 inhibitor palbociclib for five (patient 9) and three months (patient 10) (Table 1, Fig. 3A). Both patients suffered from early tumor progression (Fig. 3A).

Patient 16 with an oligodendroglioma, IDH-mutant and 1p/19q codeleted (CNS WHO grade 3) with an activating *PIK3CA* mutation was treated with the PIK3CA inhibitor alpelisib (allele frequency of *PIK3CA* mutation 33%). Only 3 days after the start of alpelisib, reactive hyperglycemia of 200 mg/dl occurred. It has been reported that the inhibition of PI3K can cause a systemic glucose–insulin feedback leading to an reactivation of PI3K signaling even in the presence of PI3K inhibitors. In a mouse model, ketogenic diet was able to suppress this mechanism and to enhance efficacy of treatment [45]. Therefore, ketogenic diet was added to alpelisib. While rapid resolution of hyperglycemia was achieved, PFS was only two months and treatment was discontinued thereafter (Fig. 3A).

A patient with medulloblastoma (patient 24) and *KMT2C* mutation (allele frequency 24%, classified as deletion mutation) was treated with the PARP inhibitor olaparib for five months until tumor progression (Fig. 3B). KMT2C has been shown to sensitize bladder cancer cells to olaparib in preclinical models [55].

Patient 27 (esthesioneuroblastoma with *NTRK3* amplification) was treated with the TKI crizotinib which was discontinued due to side effects (see below) after 6 months of therapy, although follow-up MRI showed a SD.

### Side effects and tolerability

Overall, the tolerability of therapy was good. Adverse events with fatal outcome (CTCAE grade 5) were not observed. Two patients discontinued treatment due to side effects: In patient 26 (PCP with *BRAF V600E* mutation) therapy with dabrafenib/trametinib was discontinued after three months despite a *partial response* on MRI because of three episodes of fever, some of which were sepsis-like (CTCAE grade 4) (Fig. 5B). The patient was switched to vemurafenib, which also caused serious side effects with sepsis and renal failure (CTCAE grade 4). After a brief pause in therapy, vemurafenib was resumed at low dose. With a complete response on MRI, vemurafenib was discontinued after five months of therapy (Fig. 5B).

Patient 27 (esthesioneuroblastoma with *NTRK3* amplification) who was treated with the TKI crizotinib developed an altered taste, weight loss, nausea, color perception disturbances, swelling of the right lower leg of unclear etiology, liver enzyme elevations and neutropenia (CTCAE grade 2–3). Therefore, crizotinib was discontinued after six months, although follow-up MRI showed a SD. Other AEs of CTCAE grade 3 or 4 were not observed.

## Discussion

Recent advances in gene sequencing have led to a deeper understanding of tumor biology and revealed a spectrum of clinically actionable mutations in brain tumors.

A prime example for the successful implementation of targeted therapies in brain tumors are *BRAF V600E* mutations in PCPs. *BRAF V600E* mutations can be found in 95% of all PCPs and case series report the successful exploitation of BRAF inhibitors in this entity [21, 57, 58]. In order to prevent early resistance to BRAF inhibition, the BRAF inhibitor dabrafenib can be combined with the MEK inhibitor trametinib according to protocols established for *BRAF* mutated melanoma [24, 25, 41, 68]. In line with this our index patient was by far the best treatment outcome in the entire cohort. Interestingly, this patient was treated for only 3 months with a dual BRAF/MEK inhibitory approach due to side effects and afterwards was switched to the BRAF inhibitor vemurafenib alone, which also had to be discontinued after only five months due to side effects. This patient still displays a complete response for now more than 4.5 years since start of therapy and for 4 years since therapy discontinuation. This underlines that in some cases premature discontinuation of therapy does not endanger outcome and also illustrates the central role of established key driver mutations as a therapeutic target. Nonetheless, actionable key driver mutations are rare in primary brain cancers and frequently analyses yield variants with unclear oncogenic driver potential as illustrated by the example of the two young patients with recurrent H3 K27-altered diffuse midline glioma and *FGFR1* mutations (Table 1, Figs. 3A, 5B). Diffuse midline glioma H3 K27-altered are chronically treatment-resistant tumors and new therapies are urgently needed. Based on reports on efficacy of FGFR inhibitors in brain tumors from basket trials e.g. of the FGFR1-4 inhibitor futibatinib [46], these patients were treated with FGFR1-3 inhibitor pemigatinib which in Europe is approved for the treatment of adults with locally advanced/metastatic cholangiocarcinoma with fusion or rearrangement of *FGFR2*. Unfortunately, both patients did not benefit from pemigatinib with early tumor progression after three/two months (Figs. 3A, 5B). The efficacy of pemigatinib in tumors with activating *FGFR* mutations or translocations including brain tumors will be evaluated in upcoming clinical trials including the FIGHT-207 study (NCT03822117) [47].

A better understanding of the actual activation of oncogenic signaling cascades can come from pathway analyses with phospho-immunohistochemistry stainings [69]. For example diverse genetic events including gene amplification of receptors, activating mutations of oncogenes or inactivating mutations of tumor suppressors can all induce downstream kinases that phosphorylate target proteins. Therefore, pathway analyses by IHC for phosphorylated target proteins might offer a better understanding of the actual pathway activation status via integration of different upstream pathway alterations. In the case of mTOR signaling, phospho-specific antibodies for target proteins are available but phosphorylation signals also depend on tissue quality and processing time [69]. In retrospective analyses of phase 2 and 3 clinical trials for the first line treatment of GBs, mTOR activation as assessed by IHC was identified as a biomarker to predict response to mTOR (Wick et al. 2016) or EGFR inhibitors [13, 14, 70]. In addition, parallel signaling pathways may be driving the oncogenic phenotype. To date, precision oncology predominantly focuses on molecular matching approaches using monotherapies to target one mutation with one drug, although many cancer entities are molecular heterogeneous diseases and the possibility of evasive resistance is a known phenomenon. Exceptions are the combination of BRAF and MEK inhibitors for *BRAF* mutated melanoma, PCPs and PXAs as also applied in our cohort [24, 25, 41, 68]. In line with that, targeting a larger fraction of molecular alterations yields a higher 'matching score' and can correlate with significantly improved disease control rates, PFS and OS rates [71]**. **A diagnostic bias may arise if only primary tissue is available for analysis and the disease has meanwhile relapsed. Analyses on recurrent low grade gliomas showed that in almost 50% of the cases at least half of the mutations detected in the initial tumor were lost at recurrence [72]. In our cohort in most cases the detection of the molecular alteration was performed in the recurrent tumor (Table 1). Nevertheless, therapy can be a driver for tumor evolution and most of our patients had received further treatments after detection of the molecular marker and the start of the molecular matched treatment.

Targeted therapies are undoubtedly an evolving field and new sequencing technologies offer the potential of identification of established drivers in different cancer entities. Consequently, tumor agnostic treatments selective for specific molecular alterations, regardless of tumor location and histology, are on the rise. The first tumor-agnostic drugs approved by the by the U.S. Food and Drug Administration (FDA) are the programmed death 1 (PD-1) inhibitor pembrolizumab for the treatment of patients microsatellite-instability-high (MSI-H) or mismatch-repair-deficient (dMMR) solid tumors, as well as larotrectinib and entrectinib for tumors with NTRK fusions [73]. The approval of tumor-agnostic drugs with high efficacy rates across different cancers justifies the broad implementation of (genetic) biomarker analysis also in primary brain cancer.

### Perspective

This retrospective pilot study of molecular matched therapies in brain tumors demonstrates that precision medicine based on molecular profiling is evolving in neurooncology.

Our results confirm the real world benefits of BRAF mutation-targeted therapies in brain tumors. While other bona fide driver mutations were not frequently found, the advances in molecular diagnostics over the recent years will pave the way for a more and more detailed molecular analysis of primary and recurrent tumors. Together with a broader implementation of molecular tumor boards, the increasing arsenal of molecular targeted drugs and the better understanding of tumor biology this will allow more promising off-label therapeutic interventions and promote the conduction of biomarker stratified clinical trials. The authors believe that in the not too distant future, molecular targeted therapies will be an important part for at least some subgroups of brain tumors. Critical questions down this road will be which patients to test at what stage of disease, which molecular tests/diagnostics to employ (e.g. panel vs. exome sequencing, RNA sequencing), the interpretation of these results into molecular targeted therapies, the implementation of treatment and finally the evaluation of efficacy ideally in clinical registries. Certainly, this is a formidable task for which brain tumor centers must step up and embark on a learning curve. This may be accomplished within comprehensive cancer centers by close partnerships with teams from other tumor entities, which already have made greater advances in individualized therapies to share resources and expertise.

## Supplementary Information

Below is the link to the electronic supplementary material.

Supplementary file1 Survival of brain tumor patients under molecular matched targeted therapies. A, B: Progression free survival (PFS) and overall survival (OS) of patients with at least *stable disease* (SD) and of patients with *progressive disease* (PD) treated with a molecular matched therapy. In contrast to Figure 4 all molecular matched therapies are calculated. Tick marks indicate censored patients (JPG 228 kb)

## Data Availability

All data generated or analyzed during this study are included in this published article.
