# Supplementary figures and images for: Molecular matched targeted therapies for primary brain tumors—a single center retrospective analysis
Source: J Neurooncol. 2022 Jul 21;159(2):243–59. doi: 10.1007/s11060-022-04049-w (PMC9424147; doi:10.1007/s11060-022-04049-w)

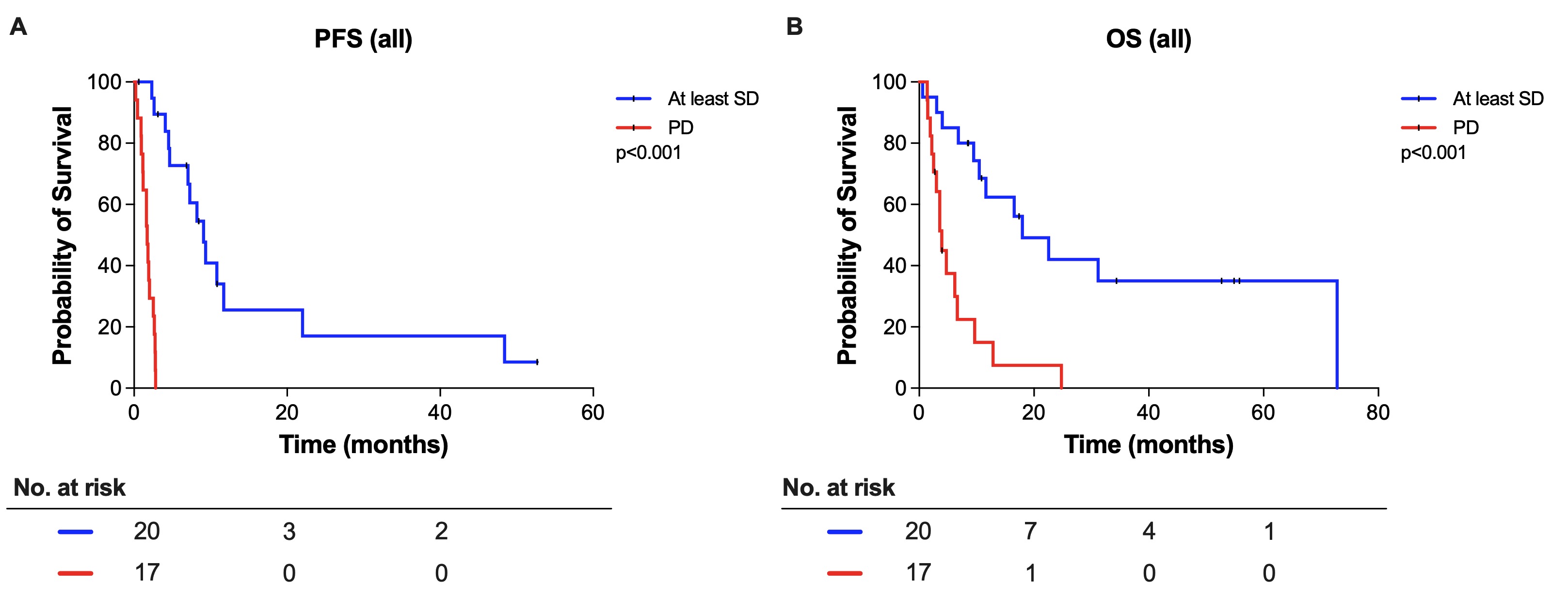

Supplement: Supplementary file 1 — Supplementary file1 Survival of brain tumor patients under molecular matched targeted therapies. A, B: Progression free survival (PFS) and overall survival (OS) of patients with at least stable disease (SD) and of patients with progressive disease (PD) treated with a molecular matched therapy. In contrast to Figure 4 all molecular matched therapies are calculated. Tick marks indicate censored patients (JPG 228 kb) [file 11060_2022_4049_MOESM1_ESM.jpg]
